# “Monkeypox: What Do You Know about That?” Italian Adults’ Awareness of a New Epidemic

**DOI:** 10.3390/pathogens11111285

**Published:** 2022-11-01

**Authors:** Francesca Gallè, Lavinia Bianco, Giovanna Da Molin, Rita Mancini, Salvatore Sciacchitano, Stefano Ferracuti, Giorgio Liguori, Giovanni Battista Orsi, Christian Napoli

**Affiliations:** 1 Department of Movement Sciences and Wellbeing, University of Naples “Parthenope”, Via Medina 40, 80133 Naples, Italy; 2 Department of Public Health and Infectious Diseases, “Sapienza” University of Rome, Piazzale Aldo Moro 5, 00185 Rome, Italy; 3 Inter-University Research Centre “Population, Environment and Health”, University of Bari Aldo Moro, Piazza Cesare Battisti 1, 70121 Bari, Italy; 4 Department of Clinical and Molecular Medicine, Sapienza University of Rome, Via di Grottarossa 1035/1039, 00189 Rome, Italy; 5 Department of Human Neuroscience, “Sapienza” University of Rome, Piazzale Aldo Moro, 5, 00185 Rome, Italy; 6 Department of Medical Surgical Sciences and Translational Medicine, “Sapienza” University of Rome, Via di Grottarossa 1035/1039, 00189 Rome, Italy

**Keywords:** monkeypox, knowledge, infection control, vaccination, vaccine hesitancy, questionnaire

## Abstract

In the course of 2022, an epidemic of monkeypox (MPX) arose worldwide. In order to assess the level of knowledge of the Italian adults regarding the new emerging disease, its prevention and their level of worrying and the acceptance of a possible vaccination, a web questionnaire was spread nationwide. A total of 1352 individuals (mean age 54.5 ± 13.4, 52.4% males) completed the questionnaire. Only 26. 7% of the sample were aware of the burden of the epidemic at the moment of the investigation; 47.1% were not able to identify the transmission route, nor the main symptoms (48.9%); and 54.2% were hesitant toward a possible MPXV vaccination, mainly because of a lack of confident in vaccines (38.5%). A low level of worrying about the disease was registered (mean score 2.3 ± 1.2 on a 5-point scale). In the regression analysis performed considering MPX knowledge as outcome, a lower level of knowledge was associated with higher age (OR 1.378, CI95% 0.998–1.904), working or studying in a nonhealthcare setting (OR 0.046, CI95% 0.033–0.066), being single (OR 0.624, CI95% 0.455–0.856) and having mass media as the main source of information (OR 0.332, CI95% 0.158–0.696). These findings indicate as of the time of this investigation, the communication about the MPXV epidemic was not effective in determining a good level of knowledge about the disease and its transmission among Italian adults. This highlights the need to improve risk communication strategies.

## 1. Introduction

The world was struck by the coronavirus disease 2019 (COVID-19) pandemic quite hard after it peaked back in 2020, and, after 2 years of cohabitation with the virus with no end in sight, the view of what we expect of (and from) new diseases has changed. Among the greatly evolving infections, in May 2022, at least 640 suspected or confirmed cases of the monkeypox virus (MPXV) infection were registered in 36 countries worldwide, with a high proportion of cases involving men who have sex with men, and on 23 July 2022, the new Monkeypox (MPX) epidemic was declared a public health emergency of international concern by the World Health Organization (WHO) [1,2,3]. At the time of writing, 73,347 cumulative cases and 29 deaths were confirmed worldwide [4].

The monkeypox virus (MPXV) is an orthopoxvirus that causes a disease with symptoms similar, but less severe, to smallpox. While smallpox was eradicated in 1979 (global eradication certificated by the WHO [5]), MPXV infection continues to occur in countries of Central and West Africa. MPX is a zoonosis, and cases are often found close to tropical rainforests where there are animals that carry the virus. Human-to-human transmission is limited. It can be transmitted through contact with bodily fluids, lesions on the skin or on internal mucosal surfaces, such as in the mouth or throat, respiratory droplets and contaminated objects [6], but a vertical route of transmission (i.e., mother to fetus) has also been reported [7], and there is rising concern toward the possibility of sexual transmission [8]. Symptoms include fever, an extensive characteristic rash and usually swollen lymph nodes, but also intense headache, back pain, myalgia and an intense asthenia [7,9]; MPXV infection is generally self-limiting, and symptoms usually resolve within 14–21 days [7]. MPX is usually characterized by a lower case-fatality ratio (CFR) than smallpox, as its lethality has been estimated between 3.6% and 10.6% depending on the subtype (West African Clade or Central African Clade) [10].

According to the experts, COVID-19 can coexist with MPXV, which can increase the risk of fatality, as it makes the body more vulnerable, and can add a burden to the already deteriorating conditions of healthcare systems due to COVID-19 [11]. Furthermore, the coemergence of this new virus might potentially increase the anxiety and worry levels due to the COVID-19 pandemic across the different sectors of the public [12,13].

It has been reported that the authorities and the public are unaware of the main characteristic of MPX [11], even considering that the younger generation has easy access to information on monkeypox that is documented on the internet and that the older generation can extrapolate information from its own history of human chickenpox infection [14]. For instance, various studies have shown a low level of knowledge even among university students [14,15].

It is evident that, to prevent the further spread of MPXV and manage the current outbreak, communication about MPX-related risks and their prevention, identification and management is crucial [11], especially when attempting to change attitudes and behavior [15]. This involves raising public health awareness regarding the spread of disease, its symptoms and preventative measures, along with specific targeting toward the most vulnerable communities and the collaboration with healthcare professionals, such as those specialized in sexually transmitted diseases, or civil society organizations [11]. In particular, risk communication should be able to identify public perceptions and address rumors and misinformation as soon as feasible [11]. In fact, an important dissemination of incorrect information by both digital and traditional media has been reported, making it difficult for the expert commentary of healthcare professionals or public health services to be heard [16]; the spread of this information can result in negative consequences, such as abstaining from adhering to appropriate health behaviors [15]. These false claims include, for example, that MPXV was created in a laboratory or that there is a link between COVID-19 vaccines and MPX prevalence [16].

The aims of this study are (i) to evaluate the level of public knowledge regarding the MPX at a national level in Italy in order to identify the gaps that need to be covered by future awareness campaigns regarding MPX and its prevention measures, as well as to counteract any fake news already being disseminated; and (ii) to identify the target population for these information campaigns.

## 2. Materials and Methods

This cross-sectional study was performed during the period of July–August 2022 in Italy through use of a web-based questionnaire.

At that time, the total number of MPXV infections diagnosed worldwide was 16,016, and the number of deaths was 5 [17].

The study does not report any experimental data on human samples, and was performed in agreement with the World Medical Association Declaration of Helsinki. Moreover, it was approved by the scientific institutional review board of the Italian Inter University Research Centre “Population, environment and health” (CIRPAS) (approval number 2707_2022).

### 2.1. Participants and Setting

Italian adults (≥18 years old) were enrolled through social media-based recruitment through use of the main web-based means of communication (i.e., mailing lists, WhatsApp, Facebook, Instagram). No payment was provided to any participants. Participants were informed about the aim and the methodologies of the study and were asked to give their informed consent to the collection and treatment of their data before completing the questionnaire.

Considering the size of the Italian adult population (49,885,100 individuals), a sample of at least 385 participants would have been required to investigate the selected variables, assuming a response proportion of 50% and a 95% confidence level.

### 2.2. Questionnaire

The questionnaire was designed with tools already used to analyze attitudes and behaviors related to COVID-19 in the same population [18,19,20,21,22], which were adapted to the new disease, and previously used questionnaires on MPX [12,14,15]. Specific questions referred to smallpox and monkeypox were also added in order to explore the level of discernment of participants towards the two pathologies, their awareness regarding the current infectious risk and their attitudes toward related control measures. The questionnaire was reviewed by a group of experts, including one epidemiologist, one public health professional, one psychologist and one expert in infectious disease. Subsequently, the modified questionnaire underwent a preliminary pilot study to test the intelligibility and the reliability of the questions on a 30-person sample (data not reported). The results confirmed that the content of the questionnaire was clear to the readers. Moreover, in the same pilot sample, the Cronbach’s alpha (internal consistency coefficient) value was 0.74, showing a satisfactory reliability.

The final questionnaire was composed of two sections. The first part was aimed to record sociodemographic information such as age, gender, educational level (elementary to high school/degree or postdegree), employment status (unemployed/working/retired) and setting (healthcare/nonhealthcare), relationship status (single or widowed/engaged or married) and health information (being affected by a chronic disease or not, having suffered from COVID-19 or not and having undergone hospitalization or not for this and being vaccinated against flu and COVID-19 or not).

The second part was aimed at assessing the participants’ knowledge about smallpox and monkeypox and their attitudes toward habit change and vaccination. They were asked to recall their awareness of smallpox even before the current MPX epidemic (“Have you heard of Smallpox before the current epidemic?”, “Is Smallpox common in Italy?” and “Have you/Has someone in your family been vaccinated against Smallpox?”) and their knowledge about MPX (“How many are the cases of MPXV infections currently?”, “Can MPX cause deaths?”, “Has anyone you know contracted MPXV infection?”, “How can MPXV be transmitted?”, “How long is the incubation time of MPX?”, “What are the main symptoms of MPX?”, “How long do MPX symptoms last?”, “Which population group has a higher risk of severe MPX?”, “Is a MPX vaccine available?”). Furthermore, participants were asked to report their main information source about MPX choosing among traditional mass media, social media and their doctors. Finally, some questions were posed to investigate if they would be favorable to a possible MPXV vaccination (“Would you undergo MPXV vaccination?”) and possibly to report the main motivation for hesitancy (“Why are you not favorable to MPXV vaccination?”). Respondents also indicated their level of worry and change of habits related to MPX prevention through a 1–5 scale, where 1 corresponded to “not at all” and 5 to “a lot”. Those who declared no change in their habits were asked to report the reason for this.

### 2.3. Statistical Analyses

A descriptive analysis was performed on the sociodemographic and health characteristics declared by participants. Age is expressed as mean value ± standard deviation (SD), median and inter-quartile range (IQR). Categorical variables and answers are expressed as number and percentage of respondents. The level of knowledge about MPXV was defined as the sum of correct answers given to the eight questions regarding the epidemiological characteristics of the infection. A comparison in the variables investigated was performed through the chi-square test between those participants whose knowledge score was lower or equal to the median value and those who had a higher score. Those variables which significantly differed between the two groups were included in a multivariate logistic regression analysis with MPX knowledge as the outcome. The results are expressed as odds ratio (OR) and 95% confidence interval (95%CI).

A *p* value of 0.05 was assumed as the significance level. Analyses were performed through the IBM SPSS version 27 software for Windows (IBM Corp., Armonk, NY, USA).

## 3. Results

The demographic and health characteristics of participants are shown in Table 1.

The sample shows a mean age of 54 years, a quite equal gender distribution and great proportions of individuals highly educated and employed, mainly in a nonhealthcare settings. The majority of the participants had or had been in a stable relationship and were affected by a chronic disease, had been affected by COVID-19 and were vaccinated against flu and COVID-19.

Table 2 reports the answers given by participants to the questions regarding smallpox and MPXV.

About three quarters of the sample declared that they had heard of smallpox before the current MPXV epidemic. However, only 41.3% thought that smallpox is not currently spreading in Italy, a percentage even lower than that of participants vaccinated (52.4%) or who had a family member (96.2%) vaccinated against the same disease. As for the questions regarding specifically monkeypox, only a small proportion of participants were aware of the burden of the epidemic at the moment of the investigation, even regarding the related deaths, and a very small portion knew someone who contracted the disease. The majority of the sample were not able to identify either the transmission route, the main symptoms or the length of the incubation and symptomatology, but immunosuppressed individuals and children were identified as categories at risk of developing severe forms of MPX by nearly the half of the respondents. About 48% of the sample were convinced that a specific vaccine against MPXV was available at the time of the investigation. The mean total score attributed to MPX knowledge was low. Traditional mass media were reported as the main source of information about MPX by the greatest portion of the respondents.

Fewer than half of the sample were favorable to vaccination. The main reason reported by those who declared their hesitancy was the lack of trust in vaccines. The level of reported worry and habit change was even lower in the sample, and the main reason for not having changed habits was the ignorance toward the risky behaviors.

Table S1 shows the differences found in demographic and health features between the participants grouped by their level of MPX knowledge.

Those who showed a higher level of knowledge were younger, more educated and occupied—especially in healthcare setting—engaged or married, less affected by chronic diseases or COVID-19 and less vaccinated against Smallpox than those who had a lower knowledge score. The group which showed better knowledge reported the knowledge of people who were affected by MPX infection and referred to information sources other than traditional mass media to a greater extent than did the other group. Respondents who had a higher knowledge score were also more favorable to undergoing MPXV vaccination, reporting the lack of risky behaviors as main reason for unwillingness, were more worried about MPXV infection and were keener to change their habits accordingly, if any, than those who had a lower score.

The results of the regression analysis performed by using the knowledge score as outcome and the demographic and health features of respondents as independent variables are reported in Table 3.

The analysis showed that a lower level of knowledge about MPX was associated with higher age, working or studying in a nonhealthcare setting, being single and having mass media as the main source of information.

## 4. Discussion

This study aimed to assess through a web-based investigation the awareness of Italian adults toward the emerging monkeypox disease. Our results show a low level of knowledge about MPX and related prevention measures in the sample examined. In fact, the majority of the sample were not able to identify the epidemiological characteristics of the disease. This is particularly worrying, considering that public engagement is vital for the success of strategies for the prevention, control and treatment of the possible outbreaks [7] and that the MPXV infection has been recognized as a risk deserving education, awareness and prevention (especially in the sexually active population, but not only) [8].

In with similar studies [7,12], the median knowledge value from the questions assessing respondents’ knowledge regarding MPX was used as a cutoff point to categorize the respondents’ knowledge scores into high or low and try to correlate them with other variables. In the regression analysis, higher age, working or studying in a nonhealthcare fields and being single were the sociodemographic variables associated with lower knowledge about MPX. It must be underlined that some of these factors are also risk factors for the disease. Starting with higher age, this is in contrast with the results of other studies, which showed that age appeared to have a significant association with better knowledge among older participants about MPX source, signs/ symptoms, transmission, prevention and treatment [7,14,15].

Students and/or healthcare workers (or in any case respondents who work in healthcare setting) represent fewer than 45% of the study population, which is in line with the results of other surveys [14], whereas in other studies, medical and dental students prevailed in the study sample [15]. In any case, the literature shows that participants from medical colleges and healthcare workers have a better knowledge score than do those studying or working in nonhealthcare settings [23], which is in line with our findings [7,14], even if some studies did not find a statistically significant difference between the mean MPX knowledge score among medical and nonmedical students [15]. Additionally, medical students are also less likely to believe in conspiracy theories compared to those in other health schools/faculties [15].

This finding is an improvement from the preliminary results published by Riccò et al. [10], as they found that fewer than one-fifth of Italian physicians, due to substantial uncertainties and knowledge gaps on MPX, were confident in being able to properly recognize incident cases during their duties; the main explanation behind this was a lack of both theoretical (did not receive a specific university-level formation on this pathogen) and practical education (respondents born in a “Variola-free” world) [10]. Considering that their sampling was done during the month of May 2022, and even taking into consideration that their subset cannot be directly compared to the overall population, it is very promising that our sampling, started in July, showed better levels of knowledge for respondents who study or work in a healthcare setting [10].

As for the education, the majority of the participants had a university degree or a postdegree title, which is in line with the results of other surveys [12].

Moreover, mass media were also found to be related with lower knowledge. In our sample, the main source of information about MPX was TV/radio/newspapers (85.6%), whereas other studies reported social networks as the main source [7,12,14]; other sources of information reported are official local and international sources, other websites and scientific journals, family or friends and healthcare providers [7,12,24]. The influence of information sources on the level of knowledge is particularly relevant, as it has been shown that higher knowledge scores are achieved by those who sourced from healthcare professionals, research articles and books compared to those who sourced information from the media, family and friends [7].

Furthermore, a low level of worry about the transmission of MPXV infection was found. In fact, the level of reported worry and habit change was registered in less than half of the sample of this study, indicating either a complete lack or very little worry toward MPX, whereas others registered that more than half of respondents were more or less worried, going from a somewhat low perceived potential threat (for instance, well below other infectious diseases) to the perception of MPX as a dangerous and virulent disease [8,12,24]. Moreover, in our sample, the main reason for not having changed habits was the ignorance toward risky behaviors, whereas in other studies there emerged a very clear belief that the MPX warranted a rapid call for respiratory and contact precautions [12]. Generally speaking, in the literature, mild anxiety levels have been reported, with particular worry toward the possibility of progression of the disease into a global pandemic and all its consequences (for instance, national lockdowns and domestic and international flight shutdowns) [12,24]. The literature also reports other worries from the monkeypox disease, such as disfiguring scarring, risks to children and pregnant women, risks of travel restrictions or requirements of additional mandatory vaccines [12].

Regarding the last point, fewer than the half of our sample were favorable to vaccination, mostly because of the lack of trust in vaccines; other studies, however, reported that about half of their respondents were in favor of vaccination [10,12], especially for immunodeficients, elderly, young children, chronically ill patients and healthcare workers [12].

Respondents who had a higher knowledge score had higher odds of supporting vaccination, which is similar to what is found in the literature, as Temsah et al. [12] registered a greater will to be vaccinated in those with greater interest in reading and searching further about MPXV infection [12]. However, it is important to keep in mind that, even between those who work or study in a healthcare setting or hold a scientific background, a strong positive attitude toward vaccination is not always present, as they can be affected by knowledge gaps, misbeliefs and emotional and personal factors in similar fashion to the general population [10].

Interestingly, the literature reports that conspiracy beliefs regarding the emergence of viral infections, such as the belief that viruses are biological weapons manufactured by superpowers to take global control or that there is a secret treatment, are widely spread; however, a higher MPX knowledge score was associated with a lower possibility to embrace these conspiracy beliefs [7,15].

Regarding previous COVID-19 infection, more than half of our respondents reported having had a previous diagnosis of infection, which is higher than what is reported in other studies [12,24].

The main limitation but also the main strength of this study is related to the recruitment procedure. In fact, the use of social media allowed us to reach a wide proportion of the Italian population, but it should be considered that only individuals with access to these communication media were involved. Therefore, this could represent a selection bias and does not allow us to apply the findings of the study to the whole Italian population.

However, notwithstanding this issue related with the type of sampling, the mean age of the sample seems to account for a good age distribution considering that the mean age of the Italian population is 46.2 years [25], and only adults were involved in this study. Furthermore, it should be noted that the questionnaire did not include questions regarding the sexual orientation of participants. Given that the 2022 human MPX outbreak has predominantly occurred in the gay, bisexual, and the men-who-have-se-with-men population, it might be possible that individuals with these sexual orientations have a better knowledge about this disease than do those in the general population, and this aspect should have been investigated in depth. Moreover, the sample was composed of a great proportion of individuals studying or working in the healthcare setting, which does not reflect the real situation in Italy. This was probably due to a higher spread of the questionnaire through social channels attended mainly by these categories and could represent another selection bias. However, this aspect seems to strengthen the importance of our findings since it is expected that healthcare personnel have a better knowledge regarding infectious diseases than do those in the general population.

Considering that the licensed therapies for human monkeypox are not yet widely available, preventive measures, including vaccination or avoiding contact with infected patients, represent the only instrument people can use to counteract this epidemic [26]. However, the employment and the acceptance of such measures requires adequate risk information and awareness.

## 5. Conclusions

Correct information is a key point of control strategies for transmittable diseases. The findings of this study indicate that as of the time of the investigation, the communication about the MPXV epidemic was not effective in determining a good level of knowledge about the disease and its transmission among Italian adults. This highlights the need for further efforts in this direction. The experience of misinformation about COVID-19 may be helpful to identifying the most effective methods to communicating the risk in Italy, especially among risk groups.

## Figures and Tables

**Table 1:** Characteristics of the sample (*n* = 1352).

Variable	
Age	
mean ± DS, median (IQR)	54.5 ± 13.4, 54 (46–68)
Gender	
*n* (%)	
males	709 (52.4)
females	643 (47.6)
Education	
elementary to high school	599 (44.3)
degree/postdegree	753 (55.7)
Occupational status	
student/unemployed	167 (12.4)
working/retired	1185 (87.6)
Study or work in healthcare setting	
*n* (%)	
no	791 (59.7)
yes	534 (40.3)
Relationship status	
*n* (%)	
single/widowed	702 (52.0)
engaged	649 (48.0)
Affected by a chronic disease	
*n* (%)	
no	486 (35.9)
yes	866 (64.1)
COVID-19	
*n* (%)	
no	265 (28.0)
yes, nonsevere	501 (52.8)
yes, severe	182 (19.2)
Vaccinated	
*n* (%)	
Flu	878 (64.9)
COVID-19	1324 (97.9)

**Table 2:** Participants’ answers regarding knowledge and attitudes toward smallpox and MPXV (*n* = 1352).

Question	Number of Respondents (%)
Smallpox questions	
Have you heard of Smallpox before the current epidemic?	
*n* (%)	
no	344 (25.4)
yes	1008 (74.6)
Is Smallpox common in Italy?	
*n* (%)	
no	558 (41.3)
yes	341 (25.2)
I don’t know	453 (33.5)
Have you been vaccinated against Smallpox?	
*n* (%)	
no	373 (27.6)
yes	709 (52.4)
I don’t know	270 (20.0)
Has someone in your family been vaccinated against Smallpox?	
*n* (%)	
no	51 (3.8)
parents	657 (48.6)
siblings	306 (22.6)
grandparents	255 (18.9)
partner	9 (0.7)
MPX questions—knowledge	
The current cases of MPXV infections are:	
*n* (%)	
about 15,000	361 (26.7)
about 500,000	200 (14.8)
about 6,000,000	219 (16.2)
I don’t know	572 (42.3)
Can MPX cause deaths?	
*n* (%)	
no	296 (21.9)
yes	555 (41.1)
I don’t know	501 (37.0)
Has anyone you know contracted MPXV infection?	
*n* (%)	
no	1321 (97.7)
yes	31 (2.3)
MPXV can be transmitted through:	
sexual intercourse	287 (21.2)
skin contacts (not sexual)	449 (33.2)
food	31 (2.3)
animals	78 (5.8)
objects	167 (12.3)
air	173 (12.8)
flies	14 (1.0)
I don’t know	637 (47.1)
The incubation time of MPX lasts:	
1–3 months	18 (1.3)
1–3 weeks	362 (26.8)
1–3 days	45 (3.3)
I don’t know	927 (68.6)
The main symptoms of MPX are:	
fever	338 (25.0)
skin eruption	600 (44.4)
muscular pain	120 (8.9)
headache	200 (14.8)
lymph node swelling	103 (7.6)
tiredness	90 (6.7)
eye symptoms	34 (2.5)
dizziness	13 (1.0)
nausea or vomit	32 (2.4)
I don’t know	661 (48.9)
The symptoms of MPX last:	
2–4 weeks	316 (23.4)
2–4 months	60 (4.4)
2–4 days	40 (3.0)
I don’t know	936 (69.2)
MPX can be severe for:	
those who have homosexual intercourses	284 (21.9)
pregnant women	191 (14.7)
immunosuppressed individuals	509 (39.2)
old adults	109 (8.4)
children	103 (7.9)
the whole population	102 (7.8)
Is a MPX vaccine available?	
*n* (%)	
no	352 (26.0)
yes	654 (48.4)
I don’t know	346 (25.6)
Total knowledge about MPX	
mean ± DS, median (IQR)	2.9 ± 2.2, 2 (1–5)
Main source of information about MPX:	
TV/radio/newspapers	1157 (85.6)
social media	108 (8.0)
doctor	87 (6.4)
MPX questions—attitudes	
Would you undergo MPXV vaccination?	
*n* (%)	
no	364 (26.9)
yes	619 (45.8)
I don’t know	369 (27.3)
If not favorable to MPXV vaccination, why?	
the disease is not severe	139 (37.7)
the disease is not common in Italy	74 (20.0)
I don’t trust vaccines	142 (38.5)
health reasons	10 (2.7)
I’m not at risk	4 (1.1)
How much are you worried about MPXV infection?	
mean ± DS, median (IQR)	2.3 ± 1.2, 2 (1–3)
How much have you changed your habits to prevent MPXV infection?	
mean ± DS, median (IQR)	1.3 ± 1.1, 1 (1–2)
If you have not changed your habits, why?	
my habits are not risky	186 (22.4)
the disease is not severe	65 (7.8)
the disease is not common in Italy	37 (4.5)
I have been vaccinated	15 (1.8)
I don’t know which habit can be risky	527 (63.5)

**Table 3:** Results of the regression analysis with MPX as the outcome.

Variable	OR (CI95%)	*p* Value
Age		0.052
≤53	1.378 (0.998–1.904)	
>53	Reference	
Gender		0.312
male	1.159 (0.871–1.542)	
female	Reference	
Education		0.245
nongraduated	0.831 (0.608–1.135)	
graduated	Reference	
Occupational status		0.187
not working	1.345 (0.865–2.092)	
working/retired	Reference	
Study or work in healthcare setting		<0.001
no	0.046 (0.033–0.066)	
yes	Reference	
Marital status		0.003
single	0.624 (0.455–0.856)	
engaged/married	Reference	
Affected by a chronic disease		0.354
no	0.846 (0.595–1.204)	
yes	Reference	
Vaccinated against:		
Flu	1.154 (0.846–1.574)	0.365
	Reference	
COVID-19	0.570 (0.211–1.543)	0.269
	Reference	
Smallpox	1.103 (0.811–1.500)	0.531
	Reference	
Having family members vaccinated against smallpox		
no	0.635 (0.288–1.400)	0.260
yes	Reference	
Knowing someone affected by MPX		0.185
no	0.468 (0.153–1.436)	
yes	Reference	
Main source of information about MPX:		
TV/radio/newspapers	0.332 (0.158–0.696)	0.004
social media	0.586 (0.237–1.449)	0.248
doctor	Reference	

## Data Availability

Data is contained within the article or supplementary material.

## Supplementary Materials

The following supporting information can be downloaded at: https://www.mdpi.com/article/10.3390/pathogens11111285/s1. Table S1: Results of the univariate analysis performed considering the level of MPXV knowledge reached by participants (701 lower or equal, 651 higher than the median value).
