# Supplementary material for: “Monkeypox: What Do You Know about That?” Italian Adults’ Awareness of a New Epidemic
Source: Pathogens. 2022 Nov 1;11(11):1285. doi: 10.3390/pathogens11111285 (PMC9696019; doi:10.3390/pathogens11111285)
Supplement: Supplementary file 1 [file pathogens-11-01285-s001.zip › pathogens-1980643-supplementary.pdf]

Table S1. Results of the univariate analysis performed considering the level of MPXV knowledge reached by participants (701 lower or equal, 651 higher than the median value).

Variable	MPX Knowledge ≤ Median Value n (%)	MPX Knowledge > Median Value n (%)	*p* Value
Age			
≤53	315 (44.9)	359 (55.1)	<0.001
>53	386 (55.1)	292 (44.9)	
Gender			
males	321 (45.8)	322 (49.5)	0.177
females	380 (54.2)	329 (50.5)	
Education			
elementary	5 (0.7)	3 (0.5)	<0.001
middle	71 (10.1)	33 (5.1)	
high school	298 (42.5)	189 (29.0)	
degree	246 (35.1)	256 (39.3)	
post-degree	81 (11.6)	170 (26.1)	
Occupational status			
not studying nor working	94 (13.4)	48 (7.4)	<0.001
studying	16 (2.3)	9 (1.4)	
working	359 (51.2)	434 (66.7)	
retired	232 (33.1)	160 (24.6)	
Study or work in healthcare setting			
no	622 (89.2)	169 (26.9)	0
yes	75 (10.8)	459 (73.1)	
Marital status			
single (never married)	59 (8.4)	77 (11.8)	<0.001
single (divorced)	217 (31.0)	138 (21.2)	
widowed	122 (17.4)	89 (13.7)	
engaged	59 (8.4)	74 (11.4)	
married	243 (34.7)	273 (41.9)	
Affected by a chronic disease			
no	172 (24.5)	314 (48.2)	0
yes	529 (75.5)	337 (51.8)	
COVID-19			
no	414 (59.1)	437 (67.1)	0.002
yes	287 (40.9)	214 (32.9)	
Vaccinated against:			
Flu	443 (63.2)	435 (66.8)	0.163
COVID-19	689 (98.3)	635 (97.5)	0.336
Smallpox	386 (55.1)	323 (49.6)	<0.001
Having some family member vaccinated against Smallpox			
no	22 (3.1)	29 (4.5)	0.204
yes	679 (96.9)	622 (95.5)	

Knowing someone affected by MPX			
no	695 (99.1)	626 (96.2)	<0.001
yes	6 (0.9)	25 (3.8)	
Main source of information about MPX:			
TV/radio/newspapers	654 (93.3)	503 (77.3)	
social media	35 (5.0)	73 (11.2)	0
doctor	12 (1.7)	75 (11.5)	
Favorable to MPX vaccination			
no	257 (36.7)	107 (16.4)	
yes	213 (30.4)	406 (62.4)	0
I don’t know	231 (33.0)	138 (21.2)	
